# Shaping Innate Lymphoid Cell Diversity

**DOI:** 10.3389/fimmu.2017.01569

**Published:** 2017-11-16

**Authors:** Qiutong Huang, Cyril Seillet, Gabrielle T. Belz

**Affiliations:** ^1^Walter and Eliza Hall Institute of Medical Research, Melbourne, VIC, Australia; ^2^Department of Medical Biology, University of Melbourne, Melbourne, VIC, Australia

**Keywords:** innate immunity, differentiation, gene expression, immune protection, innate lymphoid cell

## Abstract

Innate lymphoid cells (ILCs) are a key cell type that are enriched at mucosal surfaces and within tissues. Our understanding of these cells is growing rapidly. Paradoxically, these cells play a role in maintaining tissue integrity but they also function as key drivers of allergy and inflammation. We present here the most recent understanding of how genomics has provided significant insight into how ILCs are generated and the enormous heterogeneity present within the canonical subsets. This has allowed the generation of a detailed blueprint for ILCs to become highly sensitive and adaptive sensors of environmental changes and therefore exquisitely equipped to protect immune surfaces.

## Introduction

The importance of innate immunity has been recognized for many years. These cells play a key role in rapid responses to pathogens to protect the mucosal and external surfaces of the body. Natural killer (NK) cells and lymphoid tissue-inducer (LTi) cells are the founding members of the innate lymphoid family with the former identified more than 40 years ago. Over the past 10 years, a number of new family members have been discovered revealing an entire network of innate cells that complement the adaptive immune network. These cells went largely unrecognized for several decades which begs the question as to how they were overlooked. In this review, we summarize the current knowledge on innate lymphoid cell (ILC) differentiation and critically discuss the key challenges in the field in understanding ILC homeostatic regulation.

## Canonical ILC Subsets

**Figure 1:**
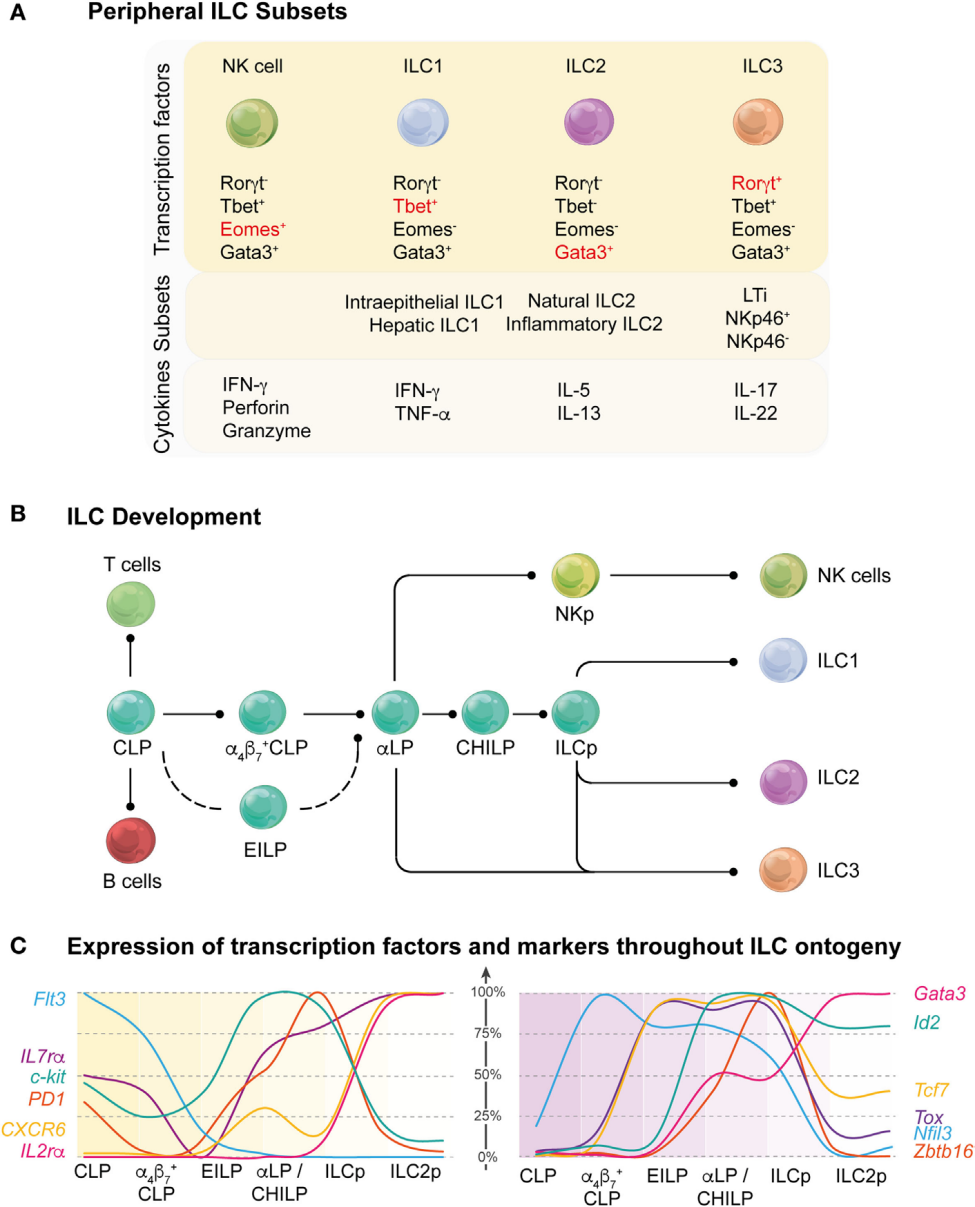
Overview of stages of innate lymphoid cell (ILC) development. **(A)** Current understanding of the regulation of peripheral ILC subsets. A variety of different transcription factors are required for the development of peripheral ILC subsets. Key transcription factors (red) are responsible for the lineage determination of the canonical ILC subsets (top panel). ILC subsets can also be further differentiated and categorized based on the organ in which they reside, functional differences, or the expression of different receptors and surface markers (middle panel). In response to activation signals, ILCs are able to produce effector molecules and cytokines to mediate an appropriate immune response (bottom panel). **(B)** Schematic showing the current understanding of ILC development from the common lymphoid progenitor (CLP) through multiple intermediary stages on their way to becoming mature ILC subsets ILC1, 2, and 3. CLP has multi-lineage potential, including T and B cell fate, but this potential is gradually lost as the progenitors differentiate into the more lineage restricted αLP. This occurs through the intermediate α4β7^+^ CLP and αLP/common helper-like ILC precursor (CHILP) progenitors or through an alternative pathway *via* the early innate lymphoid progenitors (EILPs). Within the αLP population, the natural killer (NK) cell lineage diverges from the ILC lineage and the ILC precursor (ILCp) exclusively develops into the remaining mature ILCs in the periphery. **(C)** Dynamic regulation of the surface markers (left panel) and transcription factors (right panel) throughout the ILC ontogeny. The graphs show the relative RNA expression among the different ILC progenitor stages (100% represents the highest expression for each gene detected across the six different populations).

The ILC family is divided into three major groups: group 1 ILCs (ILC1s) which includes NK cells and ILC1s that produce interferon-γ (IFN-γ) and depend on the transcription factors Eomesodermin (Eomes) and T-bet; group 2 ILCs (ILC2s) that secrete IL-5 and IL-13 and are characterized by Gata3 expression; and group 3 ILCs (ILC3s) that express the RAR-related orphan receptor, Rorγt, and includes LTi cells and multiple subsets of ILC3s capable of producing IL-17 and/or IL-22 (Figure 1A). The ILCs are distinguished from adaptive immune cells by their lack of germline rearranged antigen-specific receptors and generalized lack of lineage-specific markers normally used to distinguish B and T cells. ILCs are not thought to traffic through tissues and are often referred to as “tissue-resident” (1) but their precursors can be isolated in humans from blood (2) indicating that these cells are not completely sessile throughout their life cycle. At the very least, they transit around the body to achieve their strategic positioning close to barrier surfaces to allow them to respond rapidly to local environmental changes.

Innate lymphoid cell subsets were initially categorized based on their phenotype, function, and the key transcriptional regulators that drive their development. In many aspects, these subsets mirror CD4^+^ T cell subsets although some populations such as ILC1s have been quite difficult to position due to their lack of specific distinguishing markers (3, 4). The current classification model has served as an important framework to focus our thinking around canonical subset classifications. However, recent analyses of elegant reporters and genomic probing of individual cells has revealed that ILCs are dynamically tuned resulting in enormous heterogeneity (5–8). Potentially, this property would enable ILCs to respond to diverse stimuli in “real time.” It is widely accepted that CD4^+^ T cell subsets display extraordinary plasticity allowing them adapt to a broad spectrum of inflammatory signals, but such a program among ILCs has not been appreciated until recently. Indeed, the capacity for ILCs to exhibit a highly flexible program may be an essential element for tuning ILCs to ensure responsiveness to continuous changes in signals encountered at mucosal barriers.

Recent findings in the field have identified conceptually new ideas about how the immune system is regulated and how the innate arm might contribute to this process. For example, the ILC network forms an extensive interface between the external environment and the adaptive immune system. Their regulation is highly dynamic and relies on a highly integrated molecular signaling network, resulting in heterogeneity and plasticity. Finally, it appears to be highly complementary to the adaptive immune system providing a fail-safe mechanism for ensuring immune protection and repair processes. Excitingly, we are only just beginning to understand how this network of cells might work.

## Core Transcription Factors Establish the ILC Differentiation Framework

Innate lymphoid cells arise from the common lymphoid progenitor (CLP) through multiple intermediary stages with changes in surface expression of key surface molecules and the temporal regulation of transcriptional regulators to become mature ILC1, 2, and 3 subsets (Figure 1B). Induction of the downstream molecular program involves the induction of α_4_β_7_ which identify the α4β7^+^ CLP (9) [also called αLP1 (10)] follow by the downmodulation of Flt3 expression leading to the emergence of the α lymphoid progenitor (αLP, also called αLP2) (10, 11). While the αLP can generate all ILC subset, a subpopulation seems to have lost the ability to generate NK cells and named common helper-like ILC precursor (CHILP) (12). The distinction between the αLP and CHILP is not clear as they appear to be very highly similar in their surface marker or transcription factor expression. Finally, the induction of promyelocytic leukemia zinc finger (PLZF) in the ILC precursor (ILCp) mark the bifurcation between LTi and NK cells with the other ILC1, 2, and 3 subsets (13). Their fate is guided by lineage-determining transcription factors that are also involved in specifying different subsets of T cells. Transcription factors control multiple aspects of the development of immune cell lineages including proliferation, migration, metabolism, and effector function (Table 1). Some transcription factors play unique roles in defining the fate of early progenitors, but increasingly it is emerging that overlapping and synergistic contributions by transcription factors may be critical in setting the threshold for fate decisions and the function of an individual cell. A major challenge for the field now is to understand the combinatorial interactions between transcription factors and how they define ILC developmental choices.

**Table 1:** Requirement for different transcription factors during innate lymphoid cell (ILC) development.

Gene	Progenitors	Mature cells				Mouse phenotype
		Natural killer (NK) cells	ILC1	ILC2	ILC3	
Nuclear factor, interleukin 3 *(E4bp4)*	✓	−	−	−	−	Loss of αLP, small and fewer Peyer’s patch; normal lymph nodes, significantly reduced NK cells (14, 15)

Inhibitor of DNA binding 2 *(Id2)*	−	✓	n.d.	n.d.	n.d.	Complete loss of lymph node and Peyer’s patch formation, significantly reduced NK cells in KO and reduced IL-15 responsiveness in cKO (16, 17)

RAR-related orphan receptor gamma, RORγt (*Rorc*)	✓	−	−	−	✓	Complete loss of lymph node and Peyer’s patch formation, loss of all ILC3s (18)

B-cell lymphoma/leukemia 11B BCL11B (*Bcl11b*)	✓	−	−	✓	−	Impaired function of ILC2 *via* dysregulation of Gfi1 and IL-33 receptor (ST2) (19–21)

Thymocyte selection-associated high mobility group protein *(Tox)*	✓	n.d.	n.d.	n.d.	n.d.	Normal NKp, loss of lymph node, and Peyer’s patch formation, reduced NK cells, loss of mature NK cells (22–24)

ETS proto-oncogene1, ETS1 *(Ets1)*	✓	✓	n.d.	✓	n.d.	Reduced NK cells, hyporesponsive to IL-15 and impaired killing and degranulation, impaired ILC2 development.

T cell-specific transcriptions factor 1 *(Tcf7)*	✓	−	n.d.	n.d.	n.d.	Small Peyer’s patches, reduced NK cells in bone marrow but normal peripheral compartment (25, 26)

Promyelocytic leukemia zinc finger *(Zbtb16)*	✓	−	✓	✓	✓ [not lymphoid tissue-inducer (LTi)]	Not required in peripheral NK cells of LTi cells (13)

GATA-binding protein 3, GATA3 *(Gata3)*	✓	✓/−	✓	✓	−	Loss of GATA3 impairs NK cell maturation (27–29)

Growth factor independent 1 transcriptional repressor, GFI1 *(Gfi1)*	n.d.	n.d.	n.d.	✓	n.d.	Regulates GATA3 expression together with responsiveness *via* IL-33 receptor (ST2) (30)

*Tbx21*	−	✓	✓	n.d.	✓	Reduced NK cells, ILC1 and NCR^+^ ILC3; reduced mNK cells

*Eomesodermin*	−	✓	n.d.	n.d.	n.d.	Reduced NK cells and loss of mNK

*Pdcd1*	−	✓	n.d.	✓	n.d.	Normal secondary lymphoid tissue formation (5, 9)

✓, *required for development and/or maintenance; −, not required for development and/or maintenance; mNK cells, mature NK cells; KO, germline deletion; cKO, conditional deletion; n.d., not determined*.

The emergence of the innate cells from the CLP and the divergence of this pathway away from the adaptive lineages is an extremely controlled process that involves the coordinated actions of several transcription factors. Detailed analysis of the transcriptional landscape of the ILC development from the earliest precursor to the committed cells has revealed that regulation of the different developmental stages is highly dynamic. The sequential expression of nuclear factor interleukin 3 (NFIL3), inhibitor of DNA binding 2 (ID2), thymocyte selection-associated high mobility group box protein (TOX), and GATA-binding protein 3 (GATA3) establishes the framework for ILC differentiation (7, 9) (Figure 1C). ID2 counterbalances the effects of E proteins to direct cell choices away from T and B cell outcomes (31). Other transcription factors such as EOMES, PLZF, transcriptions factor 1 (TCF-1), and RUNX influence subset divergence. We now have new insight to the key factors that determine the fate outcome of progenitor cells under steady-state conditions. However, it still remains unclear how higher order genomic architecture establishes and maintains the differentiation program.

## The Early Regulators: A Quartet

Two major transcription factors, NFIL3 and TOX, have emerged as critical initiators of development of early αLP. Induction of NFIL3 appears to be the critical initiating step in driving the αLP toward the ILC lineage (7, 9). NFIL3 is induced in α4β7^+^ CLP, but the factors responsible for this induction have yet to be elucidated (Figures 1B,C). Concomitantly, TOX and ID2 are only expressed at low levels in the CLP but TOX expression rapidly increases in the early innate lymphoid progenitors (EILPs) while ID2 levels remains low until the late αLP and common helper ILC (CHILP) stages (12, 32–34). Nevertheless, the expression of ID2 has two patterns in ILCps; the first phase in which ID2 is expressed at low levels (and the E protein E2A is concomitantly high) and does not appear to be required for ILC development, and the second in which ID2 is strongly upregulated with concurrent downregulation of E2A and is essential for ILC lineage progression (9, 31). Indeed, while the deletion of NFIL3 blocks the development of the α4β7^+^CLP, ID2 deletion has does not appear to affect the development of the αLP. However, all cells derived from the ILC progenitor are absent in ID2^−/−^ mice suggesting that ID2 is more important in late differentiation and in maintaining the long-term identity of ILCs (16, 35). Loss of ID2 in ILCs has also been shown to repress genes belonging to the stem cell program such as *Gfi1b, Tal1, Lmo2, Gata2*, and *Hhex* (9).

Precisely how NFIL3 and TOX regulate the development of the ILC progenitors remains unclear. TOX-deficient progenitors appear to lack the expression of key factors thought to be essential for ILC development including *Gata3, Rora, Rorc, Tcf7*, and *Zbtb16* (22). Despite this, *Nfil3* expression, which could be regulated by TOX, was not found to be different from that of wild-type cells. Thus, more work will be necessary to ascertain whether NFIL3 is a direct target of TOX or not. NFIL3 has been shown to directly bind to ID2; however, it is not clear that this binding is actually responsible for the induction of ID2 (36). In ILC progenitors, ID2 expression has been found to be reduced when NFIL3 is deleted in the hematopoietic compartment. However, in mature cells, ablation of NFIL3 did not alter ID2 expression suggesting that the developmental stage of the cell influenced the interactions (37, 38). Furthermore, overexpression of either ID2 or TOX in NFIL3-deficient CLPs revealed that both transcription factors could at least partially rescue ILC development independent of NFIL3. Therefore, it is likely that the key role of NFIL3 is to promote the emergence of ILCs by induction of the expression of these two key transcription factors.

## Expression Patterning of NFIL3 and ID2 Establishes the Landscape for ILCs

Although several details of the fine tuning of NFIL3 remain unanswered, the timing and action of NFIL3 is very interesting. *Nfil3^flox/flox^* mice crossed to the *Id2^ERT2Cr^*^e^ strain generated a model in which deletion of NFIL3 could be timed relative to ID2 (9). This approach demonstrated that NFIL3 expression preceded that of ID2 but that surprisingly, deletion of NFIL3 in ID2^+^ cells either *in vivo* or *in vitro* did not affect the subsequent development of any of the ILC subsets. These findings are consistent with earlier work showing that NFIL3 was not required for the maintenance of mature NK cells (14). What was particularly unexpected, however, in the study, was the very transient nature of the NFIL3 expression which was both necessary and sufficient to promote ILC development. Indeed, only a short pulse of NFIL3 expression in the progenitors was required and it subsequently rapidly decreased as ILC progressed through each developmental stage (7, 9) and is repressed in mature cells in the periphery (15).

This pattern of short-lived expression found in NFIL3 may represent a more generalized pattern for orchestrating the complex integration of different transcriptional signals. This expression pattern has also been reported for *Zbtb16* (that encodes PLZF), another transcription factor important for ILC development but which was originally implicated in NKT cell development (39). Mature ILCs do not express PLZF; however, lineage tracing experiments revealed that 60–75% of ILCs exhibited a fluorescent imprint marking their previous expression of PLZF during development (13). In this setting, only ~5% of LTi cells and ~20% of NK cells were labeled indicating that bifurcation of these lineages from other ILC subsets occurred before the induction of PLZF. The role of the transient expression of PLZF in ILCp is not known. Using the PLZF-reporter mice, Constantinides et al. (13) also showed that PLZF is expressed only transiently. This allowed the identification of the ILCp, but PLZF was subsequently downregulated after this stage. PLZF does not appear to be absolutely required for ILCs as deletion of PLZF results in ~4-fold reduction in the number of ILCs that develop in contrast to ablation of NFIL3 which results in more than a 10-fold reduction. PLZF is expressed after NFIL3 coordinately with E2A and ID2 resulting in a stepwise progression through the early progenitor stage to generate E2A^hi^PLZF^−^ID2^lo^, E2A^hi^PLZF^+^ID2^lo^, E2A^lo^PLZF^hi^ID2^hi^, and E2A^lo^PLZF^−^ID2^hi^ expressing cells (31). In competitive situation, PLZF-deficiency appears selective, mainly affected ILC2s in the lamina propria and ILC1s in the liver (13). It was notable that ~40% of the ILC2s in this study were not labeled in these tracing experiments, so it remains possible that they could be derived from an alternative pathway that is independent of PLZF.

## Loss of IL-7R Expression: A New Progenitor or an Alternative Pathway?

The identification of the early ILC progenitor in the bone marrow, or EILP, noted for its high expression of TCF-1, has raised some questions around the linear model of ILC development (25). This EILP lacks B or T cell potential but can generate all ILC subsets including the NK cells similar to the αLP progenitor capacity, but it differs from the other precursors described as it lacks IL7Rα expression (25). One possibility is that IL-7Rα expression is lost between the αLP and the ILCp stages. Such changes could be regulated *via* posttranslational modifications though the biological relevance for such downregulation is unclear. A second possibility is that the EILP is a precursor for an alternative pathway for ILC development. EILPs express high levels of NFIL3, TOX, and TCF-1, low levels of ID2 and PLZF is almost undetectable. Interestingly, the EILPs are not affected by ID2 deletion, thus the EILPs appear to be very similar to the IL-7R-expressing αLP. A comparison between these two cell types may help to better understand the relationship between the IL7Rα^+^ and IL7Rα^−^ ILCps and define the factors that regulate IL-7R signaling in ILCs that is essential for their development. To date, TCF-1 and ID2 are known to be upregulated in EILP but additional transcriptional requirements of this progenitor have not been investigated. Therefore, whether EILP represents an intermediate stage of the ILCp, or an alternative precursor that does not fit in the current linear model of ILC development, remains an open question.

## Heterogeneity and Plasticity of ILCs are Key to Maintain Homeostasis

**Figure 2:**
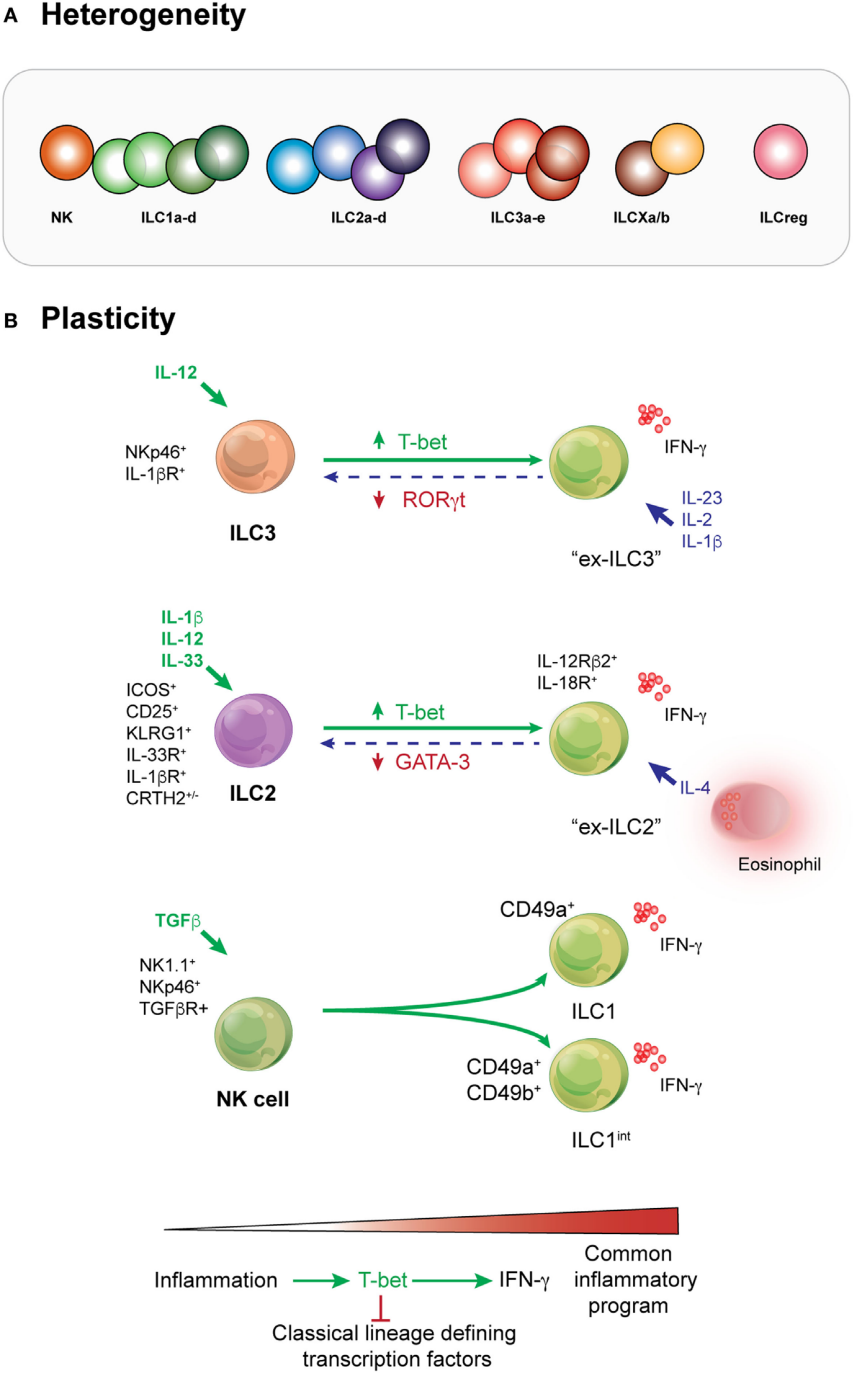
Heterogeneity and plasticity are prominent features of innate lymphoid cell (ILC) behavior across all subsets. **(A)** Deep analysis into the transcriptome of ILCs demonstrates that heterogeneity occurs within every subset. Depending on the type or intensity of the stimuli received by these cells, different molecular pathways may be activated by cells of the same subset. This results in phenotypic or functional variation and a subsequent spectrum of ILCs within each compartment. Whether additional subsets such as the proposed regulatory subset exist is yet to be fully determined. **(B)** ILC3s can adopt an ILC1-like phenotype when activated by IL-12. They are known as “ex-ILC3s.” In human cells, this pathway can be reversed by the action of IL-23, IL-2, and IL-1β. ILC2s are activated by IL-2 and IL-33. Stimulation with IL-1β primes the responsiveness of ILC2 by enhancing the expression of cytokine receptors such as IL-25R, IL-33R, and TSLP to potentiate ILC2 responsiveness and induce a significant increase in the population. Critically, however, IL-12 is essential to effect remodeling of the chromatin landscape in ILC2 allowing them to induce phenotypic changes and become more like ILC1s (ILC1-like or “ex-ILC2s”) that produce IFN-γ. Natural killer (NK) cells respond to TGF-β to form “intermediate ILC1” reflecting their acquisition of CD49a and *bone fide* ILC1. In many situations, it appears that the transcription factor T-bet is key to augmentation of the inflammatory program and concurrently represses signature transcription factors that typically define individual lineages.

Initial categorization of ILC subsets relied on cytokines and effector molecules they produced combined with signature transcription factors that appeared to be central regulators of the different subsets. The patterns found in ILCs were aligned to the categorization of CD4^+^ T cell subsets. Unlike CD4^+^ T cells, however, it quickly emerged that several ILC phenotypes did not necessarily neatly fit into the unified nomenclature coined in 2013 with subsets described as being “like” other subsets (40). The full spectrum of ILC heterogeneity has recently been unveiled using comprehensive single-cell sequencing combined with mass cytometry approaches which has allowed the field to bridge between our understanding of ILCs in mouse and man (2, 6, 41, 42) (Figure 2A). This approach revealed that potentially as many as 15 transcriptionally distinct identities could be delineated in the small intestine and that crosstalk orchestrated *via* cytokines such as IL-12 and IL-23 and microbial signals were substantially responsible for mediating this subset plasticity (6). It is undoubtable that these approaches are transforming our understanding of diversity in immune cell subsets but these data also throw up new technical and intellectual challenges in understanding how diversity arises. Historically, various surface markers have been considered to represent “lineage specificity” but we now recognize that this is seldom the case, challenging that how we interpret complex data and underlying that subsets cannot be defined exclusively by particular markers or transcription factors (43). Nevertheless, it provides a rich landscape for understanding how different stimuli affect the homeostatic balance of ILC subsets. It has been revealed that ILCs exhibit tissue-specific characteristics and this in part reflects alterations in phenotype that can be significantly regulated by responses to inflammation and infection.

The first example of ILC ability to adopt a different phenotype was the identification of ILC3s that downregulated RORγt but expressed T-bet, NK1.1, and produced IFN-γ (also known as ex-ILC3s) (12, 44, 45) under chronic stimulation and RORγt^+^ ILC3 that produced IL-17 which were found in the large intestine (46) (Figure 2B). IL-12 was found to be a key driver of this pathway. Similarly, IL-1β, together with combinations of IL-4, IL-12, and IL-33, can drive ILC2s to adopt an IFN-γ-producing phenotype called ex-ILC2 (47–49). ILC2s can be divided into two subsets: homeostatic or natural ILC2s which typically reside in barrier tissues and respond to IL-33; and inflammatory ILC2s which are generally not found in peripheral tissues at steady-state but respond to IL-25 which induces multipotency (50–53). In this setting, T-bet (encoded by *Tbx21*) was induced, whereas GATA3 was diminished. These studies highlight the variability that can occur in ILCs, particularly *in vitro* as demonstrated for ILC2s (47–49). Indeed, even NK cells can undergo this sort of transformation (54). Signaling through the TGF-β pathway can convert NK cells (CD49a^−^CD49b^+^Eomes^+^) into intermediate ILC1 (CD49a^+^CD49b^+^Eomes^+^) and *bone fide* ILC1 (CD49a^+^CD49b^+^Eomes^+/−^) within a tumor microenvironment. Strikingly, these latter ILC1 were disabled in their capacity to control local tumor growth and to prevent metastasis, while NK cells retained their ability to undertake immune surveillance (54). It is striking that once activated through these different pathways, each subset converges on an “IFN-γ-producing ILC1-like” phenotype. This suggests that this fate may represent a common outcome for multiple ILC subsets, even perhaps an essential adaptive program for all ILCs. It remains a challenge, however, to understand how these IFN-γ-producing cells arise and whether it represents a protective response or the first steps to loss of immune control (Figure 2B). Teasing this apart will require much more extensive study particularly in the context of pathogen infection, inflammation, and tumor development.

## Are More Subsets Possible?

How ILCs populate tissues after birth or maintain their presence in peripheral tissue is not clear. Our current understanding indicates that ILCs are almost exclusively tissue resident and that they do not routinely circulate throughout the body. This perception is predicated on the findings that during engraftment following irradiation, donor ILCs largely fail to replace ILCs originally found in the host (1). Instead, they depend on local proliferation to expand and replace ILCs, and it is only late in an infection or physiological disturbance that replenishment from blood-borne precursors restores the integrity of the ILC compartment. But have we got this right? These findings depend on a number of assumptions. For example, it is presumed that the transcriptional regulators, surface molecules, maturity, and frequency of relatively “mature” ILCs are also the most useful for pinpointing circulating ILCs. In addition, the high similarity of markers in murine ILCs that are comparable in man may have obscured our ability to identify circulating progenitors, or more mature cells, that are critical to maintain tissue homeostasis.

### Circulating Precursors

The first clue that programming might potentially differ between mouse and man came from Scoville et al. (55) who observed that RORγt was expressed in all human ILCs. This was in striking contrast to murine ILCs where RORγt expression was highly restricted to the ILC3 subset (18). Interestingly, CD34^+^ progenitors expressing c-kit and RORγt could generate all ILC subsets, including NK cells. These progenitors selectively resided in secondary lymphoid tissues. This helped to identify the pathway of ILC development in man; however, the involvement of these precursors remained unclear. Later, an extensive analysis of the c-kit^+^ ILCs from the blood and tissues revealed that circulating ILCp in humans exist and do not typically express many of the markers associated with mature cells but do express low levels of RORγt (2). Such cells are maintained in RORC-deficient patients and retain the potential to produce different populations of ILC except ILC3. Thus, it appears that high expression of RORγt is necessary to generate an ILC3 in both man and mouse, but these observations raise the question of whether in mice similar progenitors have been simply discounted due to their low expression of this transcription factor. However, while the role of RORγt in the development of human ILCps is unclear, transcriptomic and epigenomic analysis of circulating human ILCps revealed the upregulation of many transcription factors known to be critical for ILC development in mice such as *NFIL3, ID2, TOX, TCF7, ZBTB16*, and *GATA*3 (2). This suggests that transcriptional regulation of ILC development share common factors in human and mouse.

### Regulatory ILCs

Innate lymphoid cell subsets appear to largely mimic those defined for CD4^+^ T cells. Recent evidence suggests that this extends to the presence of regulatory ILCs. Among the ILCs, NK cells have been described to produce IL-10 (56, 57) that acts to depress B cell (58) and dendritic cell immune responsiveness (59, 60) and suppress activation. The Ohashi group (61) strengthens the notion that a regulatory subset might exist. During their evaluation of a tumor-infiltrating cell-based adoptive immunotherapy for ovarian cancer, it was noticed that a high frequency of CD56^+^CD3^−^ cells was strongly correlated with suppression of tumor-infiltrating cell outgrowth and proposed that these cells played a regulatory role. While this effect has only been tested *in vitro*, they ascertained that regulatory and conventional CD56^+^CD3^−^ ILCs exhibited high levels of the transcription factors *ID2, ZBTB16 (PLZF), RUNX3*, and *TOX*, but similar amounts of *EOMES, TBX21, GATA3, RORA*, and *AHR*, factors also shared with NK cells, ILC2s, and ILC3s. Very recently, however, Wang et al. (62) provide the first description of regulatory ILCs in mice and humans which were shown to be important in response to gut inflammation such as *Citrobacter rodentium*. This subset arises from an ID2-expressing progenitor and depends on a second inhibitor of DNA-binding protein, ID3 but not PLZF or RORγt. Intriguingly, this subset lacks expression of any of the classical transcription factors required for the early steps in development by other ILCs including NFIL3, TOX, TCF-1, GATA3, or PLZF. Thus, it remains unclear whether the early progenitor is the ILCp, or alternatively a distinct progenitor gives rise to this subset. However, like other ILC subsets they lack typical lineage markers but do express CD25 and CD90 while autocrine expression of TGF-β1 drives expansion of this IL-10^+^ subset during inflammation and results in suppression of activation of ILC1 and ILC3.

Combined, these important studies point toward the existence of a regulatory subset, but important questions still remain. For example, how and when do regulatory ILCs emerge, what other transcription factors drive this process, and do they express receptors that can be targeted to restore immune homeostasis during chronic and autoimmune diseases. If they do, this proves another avenue to unleash the protective power of ILCs either within tumors or during inflammation and in maintaining normal homeostasis to prevent autoimmunity.

## Concluding Remarks

With increasing understanding of the regulation of the ILC, we realize how extensive is their ability to adapt their microenvironment. While ILC subsets are often seen as innate counterpart of T helper cells, it may be interesting to imagine the ILCp as an innate counterpart of naive T cells. ILC subsets also to appear to be an extremely plastic population that can profoundly change their predicted responses in reaction of extracellular mediators. One important challenge will be to identify the large variety of environmental and host-derived signals they can integrate to understand the role of ILCs in the homeostasis of the tissue and during inflammation.

## Author Contributions

All authors contributed to the manuscript and read, edited, and approved the final manuscript.

## Conflict of Interest Statement

The authors declare that the research was conducted in the absence of any commercial or financial relationships that could be construed as a potential conflict of interest.
